# Attenuation Profile and Molecular Characterization of a High-Passaged Attenuated Getah Virus Strain

**DOI:** 10.1155/tbed/2787909

**Published:** 2025-11-13

**Authors:** Jing Chen, Haichao Wu, Anqi Lin, Xingbo Miu, Yuchen Li, Qiulin Zhu, Yingmei Lu, Xiaoyan Zhang, Zhibang Zhang, Kai Li, Pengcheng Li, Taotao Yang, Yuli Hu

**Affiliations:** ^1^School of Life Sciences and Resource Environment, Yichun University, Yichun 336000, Jiangxi, China; ^2^Laboratory of Animal Pathogenic Microbiology, Yichun University, Yichun 336000, Jiangxi, China; ^3^Sinopharm Animal Health Corporation Ltd., Wuhan 430075, Hubei, China; ^4^College of Bee Science and Biomedicine, Fujian Agriculture and Forestry University, Fuzhou 350002, Fujian, China

**Keywords:** amino acid substitution, attenuation, Getah virus, virulence

## Abstract

In recent years, the prevalence of epizootic diseases caused by Getah virus (GETV) has surged in China, raising significant concerns for animal health and posing a potential threat to public health. This study aims to systematically compare the phenotypic and genotypic characteristics of a high-passage attenuated GETV strain (HuN1-P230) with its virulent parental strain (HuN1), elucidating the molecular changes associated with the attenuation process. The HuN1-P230 strain exhibited enhanced replication kinetics, higher viral titers, and a small-plaque phenotype in cell cultures compared to HuN1. Notably, pregnant mice inoculated with HuN1-P230 displayed a 100% survival rate among neonates, in stark contrast to the complete absence of live births observed with the parental HuN1 strain, indicating a highly attenuated virulence phenotype. Furthermore, challenge experiments demonstrated that HuN1-P230 conferred complete protection against the virulent HuN1 strain. Genomic comparative analysis revealed that HuN1-P230 harbored 26 nucleotide mutations relative to HuN1, including 11 silent mutations and 15 amino acid substitutions. Structural analysis of the GETV spike protein indicated that the observed antigenic differences were closely linked to amino acid substitutions located on the viral surface. These findings suggest that the phenotypic changes observed during GETV attenuation are closely associated with specific genetic modifications, providing critical insights into the molecular mechanisms underlying viral attenuation and highlighting the potential of HuN1-P230 as a vaccine candidate.

## 1. Introduction

Getah virus (GETV), an arthropod-borne virus (arbovirus) with a broad host range, has been documented to infect a wide variety of vertebrate species, including horses, pigs, cattle, foxes, and humans [1]. Recognized as a significant zoonotic pathogen, GETV exhibits considerable pathogenicity across multiple mammalian hosts. In piglets, GETV infection manifests as pyrexia, anorexia, ataxia, tremors, and increased mortality, while infected pregnant sows often experience severe reproductive complications, including abortion, stillbirths, fetal mummification, and irregular estrus cycles [2, 3]. In equines, clinical signs of GETV infection include fever, urticaria, and edema of the hind limbs [4, 5], whereas infected foxes present with symptoms such as fever, anorexia, neurological disturbances, and high mortality rates [6]. In recent years, the incidence of GETV-associated epizootic diseases has risen significantly in China [2–4, 6–9], underscoring its potential as a growing threat to animal health and, by extension, public health. However, there are currently no commercially available GETV vaccines, highlighting the necessity to develop effective vaccine candidates against GETV.

GETV, a member of the Alphavirus genus within the Togaviridae family, is a linear, single-stranded, positive-sense RNA virus [10]. Its genome, ~11.7 kilobases in length, contains two open reading frames that encode nonstructural and structural polyproteins. These polyproteins are subsequently cleaved into four nonstructural proteins (nsP1-4) and five structural proteins (C, E3, E2, 6K, and E1) [1]. GETV virions exhibit icosahedral symmetry and are enveloped particles with a diameter of ~70 nm [10]. Each virion consists of a nucleocapsid core surrounded by a lipid bilayer embedded with glycoprotein spikes [11, 12]. These spikes are composed of E1-E2 heterodimers, with each virion displaying 80 trimeric glycoprotein spikes on its surface [11, 12]. The nucleocapsid core is formed by 240 copies of the capsid protein, which encapsidate the viral genome [11, 12].

Amino acid residues in viral proteins have been identified as critical determinants of alphavirus virulence [13–16]. For instance, Wang et al. [17] demonstrated that the K253R substitution in the E2 protein of GETV enhances viral infectivity in mammalian cells while attenuating virulence in murine models. In addition, mutations at the C-terminal of the nsP2 gene (K648A and R649A) have been shown to impair nuclear transport of nsP2, thereby reducing viral virulence by disrupting GETV's ability to antagonize the host innate immune response [18]. Despite these findings, the mechanisms underlying GETV attenuation and virulence remain poorly understood.

In this study, we aimed to address this knowledge gap by serially passaging a virulent wild-type GETV strain (HuN1), isolated from the cerebral tissues of an infected piglet, in PK-15 cells for 230 generations to generate an attenuated strain (HuN1-P230). We conducted a comprehensive analysis of the biological characteristics, genomic sequences, and amino acid residues of the major structural proteins of HuN1-P230.

## 2. Materials and Methods

### 2.1. Cells, Viruses, and Mice

PK-15 (GDC0061) and BHK-21 (GDC0010) cells purchased from CCTCC (https://cctcc.whu.edu.cn/) were maintained in Dulbecco's Modified Eagle Medium (DMEM; Gibco, USA) supplemented with 6% newborn calf serum (NBCS; Gibco, USA) and 1% penicillin-streptomycin (P&S; ShareBio, China), and incubated at 37°C in a 5% CO_2_ atmosphere.

The HuN1 strain of GETV, previously isolated from the cerebral tissues of a piglet [3], was used as the virulent strain in this study. The HuN1 strain had undergone six passages in PK-15 cells prior to use. To generate the attenuated strain, HuN1-P230, the virulent HuN1 strain was serially passaged an additional 230 times in PK-15 cells.

Specific pathogen-free (SPF) ICR mice were obtained from Hunan SJA Laboratory Animal Co., Ltd. (Changsha, China). Mice were housed in individually ventilated cages. All animal experiments were conducted in compliance with the guidelines of the Institutional Animal Care and Use Committee (IACUC) of Yichun University (Permit Number: JXSTUDKY2025050) and adhered to relevant ethical standards.

### 2.2. Growth Curve Analysis

Monolayer cultures of PK-15 cells were seeded in 6-well plates and infected with HuN1 or HuN1-P230 at a multiplicity of infection (MOI) of 0.01. After 1 h of incubation at 37°C, the viral inoculum was removed, and the cells were washed with phosphate-buffered saline (PBS). Fresh DMEM supplemented with 2% NBCS and 1% P&S was added, and the cultures were maintained at 37°C in a 5% CO_2_ atmosphere. Cell samples were collected at predetermined time points (12, 24, 36, 48, 60, 72, 84, and 96 h post-inoculation) for viral titration.

Viral titers were determined using the 50% tissue culture infective dose (TCID_50_) assay. Briefly, PK-15 cells in 96-well plates were infected with tenfold serial dilutions of the virus. Each dilution was tested in eight replicate wells (100 µL per well). After 4 days of incubation at 37°C in a 5% CO_2_ atmosphere, cytopathic effects (CPE) were assessed by microscopy. The viral titer was calculated using the Reed-Muench method.

### 2.3. Plaque Size Determination

Monolayer cultures of BHK-21 cells in 6-well plates were infected with HuN1 or HuN1-P230 at titers of 200, 100, 50, and 10 TCID_50_ per 0.1 mL. After 1 h of incubation at 37°C, the viral inoculum was removed, and the cells were washed with PBS. The cells were then overlaid with 2 mL of fresh DMEM supplemented with 2% NBCS, 1% P&S, and 1% low-gelling-temperature agarose, and maintained at 37°C in a 5% CO_2_ atmosphere. At 4 days postinfection (dpi), the cells were fixed with 4% paraformaldehyde and stained with 0.5% crystal violet. Plaque sizes were measured using slide calipers.

### 2.4. Pathogenicity in Pregnant Mice

To evaluate the pathogenicity of HuN1, HuN1-P50, HuN1-P130, and HuN1-P230, pregnant mice at 1 day of gestation were intravenously inoculated with 0.1 mL of viral suspension (10^6^ TCID_50_/mL). Control mice received an equivalent volume of saline via the same route. Each group consisted of six mice. Clinical manifestations in pregnant mice were monitored daily until the estimated due date. The body weight of neonatal mice was recorded every 4 days for 13 days post-birth. At the end of the experimental observation period, all mice were euthanized via cervical dislocation following 2 h of inhalation anesthesia with 3% sevoflurane.

### 2.5. Immune Protection in Pregnant Mice

To assess the immune protection conferred by HuN1-P230 against the virulent HuN1 strain, adult female mice were intravenously immunized with 0.1 mL of HuN1-P230 suspension (10^6^ TCID_50_/mL). Control mice received saline under the same conditions. After 13 days of immunization, the mice were mated. Pregnant mice at 1 day of gestation were intravenously challenged with 0.1 mL of HuN1 suspension (10^6^ TCID_50_/mL). Clinical manifestations were monitored daily until the estimated due date. At the end of the experimental observation period, all mice were euthanized via cervical dislocation following 2 h of inhalation anesthesia with 3% sevoflurane.

### 2.6. Full-Length Genome Sequencing and Alignment

Viral RNA was extracted from the supernatants of HuN1, HuN1-P50, HuN1-P130, and HuN1-P230 cultures. Reverse transcription was performed using the RevertAid First Strand cDNA Synthesis Kit (Thermo Fisher Scientific, USA) according to the manufacturer's instructions. PCR amplification was conducted using pfu DNA polymerase (Vazyme, China), and the resulting products were cloned using the pEASY-T1 Cloning Kit (TransGen Biotech, China) as previously described [3]. The cloned products were sequenced by BioSune (Shanghai, China). Sequence alignment and analysis were performed using MEGA 6.06 software [19]. Phylogenetic analysis was performed using the maximum-likelihood (ML) method in MEGA version 6.06. The Tamura-Nei substitution model, incorporating gamma-distributed rate heterogeneity, was employed to construct the ML tree. Branch support was assessed with 1000 bootstrap replicates.

### 2.7. Protein Structure Analysis

Protein models were visualized using PyMOL software (Molecular Graphics System, Version 3.0, Schrödinger, LLC; https://pymol.org) with the Cryo-EM structure of GETV (PDB file: 7WC2) [12].

### 2.8. Preparation of Antisera and Serum Neutralization (SN) Test

In the preparation of immune sera, mice were injected intraperitoneally with 0.3 mL viral suspension for the HuN1 and HuN1-P230 strains at a concentration of 10^6^ TCID_50_/mL (*n* = 3 per group). Blood samples were collected via orbital bleeding 14 days postimmunization. A virus neutralization test utilizing the microtiter method was conducted to quantify the neutralizing antibodies against the HuN1 and HuN1-P230 strains. The antisera for both strains were heat-inactivated at 56°C for 30 min. Subsequently, 50 μL of inactivated antiserum was serially diluted twofold and mixed with a viral suspension containing 200 TCID_50_/0.1 mL. Each dilution was tested in duplicate wells. After incubation at 37°C for 60 min, the mixtures were transferred to a 96-well microplate containing PK-15 cell cultures. The cells were then incubated at 37°C under 5% CO_2_ for 4–5 days. The neutralizing antibody titer was defined as the reciprocal of the highest serum dilution that completely inhibited CPEs.

### 2.9. Statistical Analysis

All data are presented as mean ± standard deviation (SD). Statistical analyses were performed using GraphPad Prism software (Version 9.0.0). Differences of plaque size between experimental and control groups were evaluated using Student's *t*-test, with effect sizes (Cohen's *d*) considered as small, medium, or large (<0.5, 0.5–0.8, and >0.8, respectively). For comparisons of neonatal mouse outcomes, group-wise comparisons (HuN1, HuN1-P50, HuN1-P130, HuN1-P230 vs saline controls) were performed using one-way ANOVA followed by Dunnett's post hoc test for multiple comparisons. Longitudinal analyses (days 1–13 post-birth) comparing HuN1-P230, HuN1-P130, and control groups were conducted using two-way ANOVA with Tukey's post hoc test for multiple comparisons. Statistical significance was defined as *p* < 0.05 (*⁣*^*∗*^), *p* < 0.01 (*⁣*^*∗∗*^), *p* < 0.001 (*⁣*^*∗∗∗*^), and *p* < 0.0001 (*⁣*^*∗∗∗∗*^).

## 3. Results

### 3.1. Replication Kinetics of HuN1-P230 in Cell Culture

**Figure 1:**
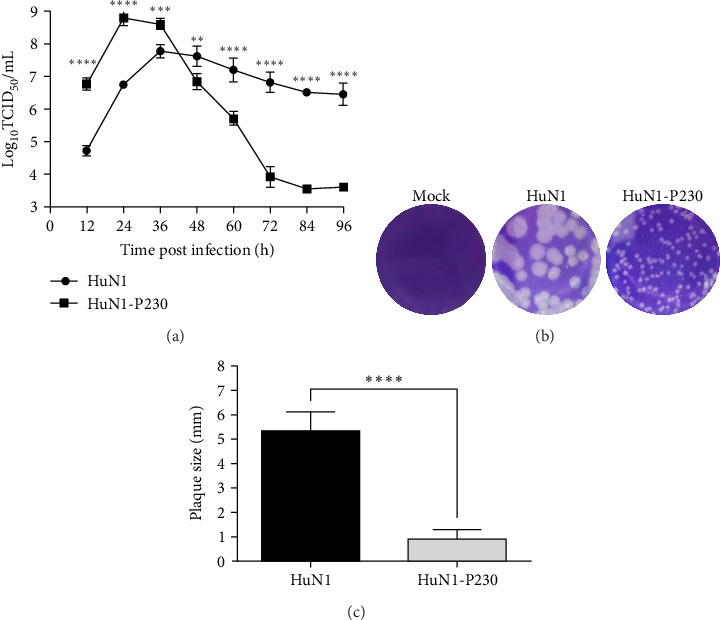
Comparative replication kinetics and plaque morphology of GETV strains HuN1 and HuN1-P230 in vitro. (A) Viral growth curves in PK-15 cells infected at an MOI of 0.01. Viral titers were determined by TCID_50_ assay at indicated time points (*n* = 3). (B) Representative plaque morphology in BHK-21 cells at 5 dpi, stained with crystal violet. (C) Quantitative analysis of plaque diameters. Data represent mean ± SD from three biological replicates. Statistical significance: *⁣*^*∗*^*p* < 0.05, *⁣*^*∗∗*^*p* < 0.01, *⁣*^*∗∗∗*^*p* < 0.001, *⁣*^*∗∗∗∗*^*p* < 0.0001 (Student's *t*-test).

To evaluate the replication dynamics of the virulent HuN1 strain and its attenuated counterpart, HuN1-P230, viral replication kinetics were analyzed in PK-15 cells. The viral titer of HuN1-P230 peaked at 24 h postinfection (hpi), reaching 10^8.79^ TCID_50_/mL, whereas the HuN1 strain reached its maximum titer of 10^7.78^ TCID_50_/mL at 36 hpi (Figure 1A). Prior to 36 hpi, HuN1-P230 exhibited significantly higher replication titers compared to HuN1. However, after 36 hpi, cells infected with HuN1-P230 underwent rapid detachment and cell death, leading to a sharp decline in viral titers.

Plaque morphology analysis in BHK-21 cells revealed that HuN1 formed plaques with an average size of 5.33 ± 0.75 mm, significantly larger than those of HuN1-P230, which measured 0.97 ± 0.32 mm (Cohen's *d* = 6.313, 95% CI: 5.35–7.27, *p*  < 0.0001, Figure 1B,C). These findings suggest that HuN1-P230 exhibits a small-plaque phenotype, a characteristic often associated with viral attenuation. The paradox of enhanced replication yet reduced plaque size maybe attributed to the altered cell-to-cell spread efficiency of the HuN1-P230.

### 3.2. Pathogenicity of HuN1-P230 in Pregnant Mice

**Figure 2:**
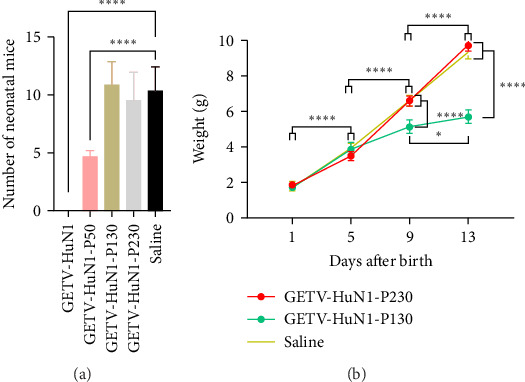
Pathogenicity of GETV HuN1 and its passaged strains in pregnant mice. (A) Number of neonatal mice following inoculation with HuN1, HuN1-P50, HuN1-P130, and HuN1-P230. Data were analyzed by one-way ANOVA. (B) Body weight of neonates from the HuN1-P130 and HuN1-P230 groups compared to negative controls. Data were analyzed by two-way ANOVA. Data represent mean ± SD from three biological replicates. Statistical significance: *⁣*^*∗*^*p* < 0.05, *⁣*^*∗∗*^*p* < 0.01, *⁣*^*∗∗∗*^*p* < 0.001, *⁣*^*∗∗∗∗*^*p* < 0.0001.

The pathogenicity of HuN1, HuN1-P50, HuN1-P130, and HuN1-P230 was assessed in pregnant mice at 1 day of gestation. Mice inoculated with the virulent HuN1 strain exhibited severe pathogenic effects, including complete embryonic resorption and no live births (Figure 2A). In contrast, inoculation with HuN1-P50 resulted in significantly fewer live births compared to the negative control group (Figure 2A). Notably, all mice inoculated with HuN1-P130 and HuN1-P230 successfully gave birth, with almost a 100% survival rate among neonates (Figure 2A). Furthermore, no significant differences in body weight were observed between neonates from the HuN1-P230 group and the negative control group during the 13-day observation period (Figure 2B). However, neonates from the HuN1-P130 group exhibited a significant reduction in body weight at 9 and 13 days postpartum compared to controls (Figure 2B). These results demonstrate that HuN1-P230 is significantly attenuated in virulence compared to the wild-type HuN1 strain.

### 3.3. Immune Protection Conferred by HuN1-P230 Strain in Pregnant Mice

To evaluate the immune protection provided by HuN1-P230 against the virulent HuN1 strain, vaccination and challenge experiments were conducted. Control pregnant mice inoculated with HuN1 exhibited severe pathogenic effects, including complete embryonic resorption and no live births. In contrast, all mice immunized with HuN1-P230 successfully gave birth, achieving a 100% survival rate (63/63) among neonates. These findings indicate that HuN1-P230 confers complete protection against the virulent HuN1 strain.

### 3.4. Genomic Analysis of HuN1-P230

**Figure 3:**
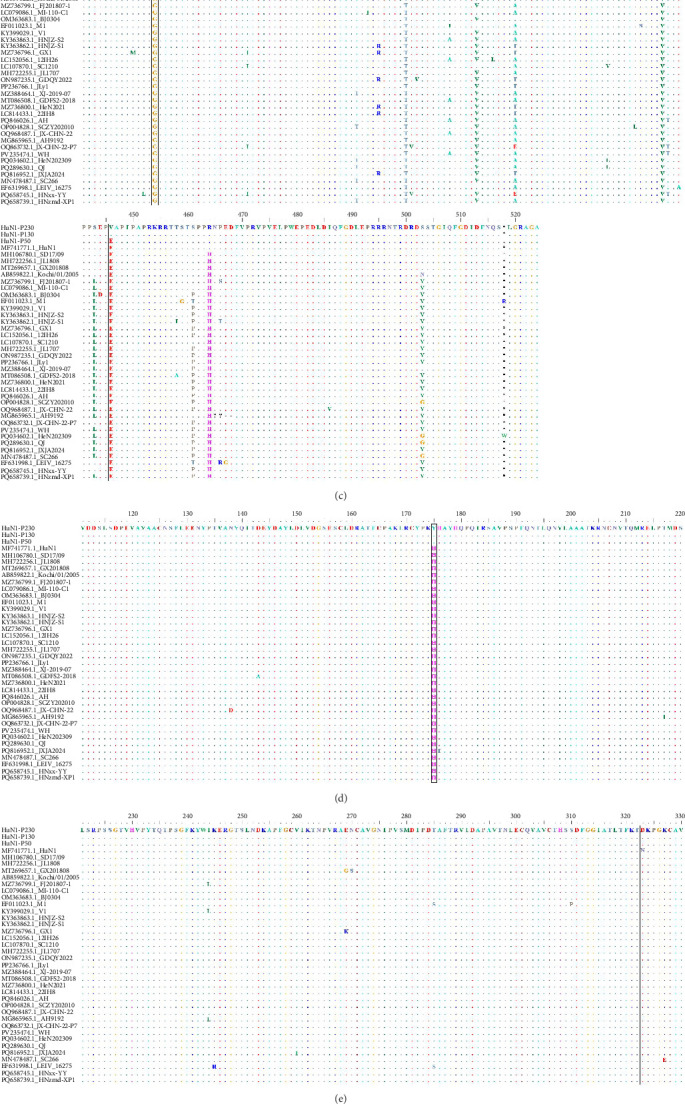
Amino acid sequences alignment of GETV strains. Sequences in boxes show amino acids at residues 214, 220, and 450 in the nsP1 (A), 650 in the nsP2 (B), 344 and 446 in the nsP3 (C), 175 in the nsP4 (D), 323 in the E1 (E), and 4, 173, 178, 214, 222, 232, and 322 in the E2 (F). Dots represent identical amino acids with consensus sequences.

Comparative genomic analysis revealed distinct nucleotide polymorphisms and corresponding amino acid substitutions between the virulent HuN1 strain (GenBank accession no. MF741771) and its passaged derivatives, HuN1-P50, HuN1-P130, and HuN1-P230 (GenBank accession nos. PV904260–PV904262). The viral genome exhibited 26 nucleotide mutations, all localized within protein-coding regions, which translated to 15 nonsynonymous amino acid changes. These variations were distributed across both structural and nonstructural proteins: seven in the E2 glycoprotein, three in nsP1, two in nsP3, and single substitutions in nsP2, nsP4, and E1 proteins (Table 1, Figure 3). Notably, the majority of these mutations (11/26) represented silent mutations that did not alter the amino acid sequence (Table 1).

**Figure 4:**
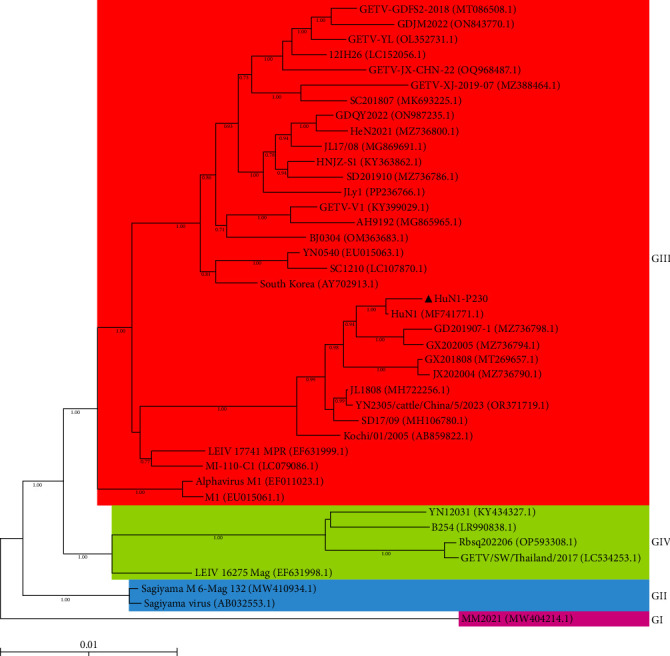
Phylogenetic analysis of GETV HuN1-P230 in the context of global strains. A maximum-likelihood tree was constructed using aligned complete genome sequences (11,462 nt). The phylogenetic reconstruction was performed in MEGA 6.06 under the Tamura-Nei model with gamma-distributed rate variation. Branch support values (from 1000 bootstrap replicates) greater than 70% are displayed at the nodes. The HuN1-P230 strain investigated in this study is indicated by a black triangle (▲). Scale bars represent the number of nucleotide substitutions per site.

Phylogenetic analysis was conducted based on the full-length nucleotide sequences of 40 GETV reference strains retrieved from NCBI. The results revealed that these GETV strains can be categorized into four distinct groups (I–IV). The HuN1-P230 strain clustered within Group III, which comprises isolates originating from China, Japan, South Korea, and Mongolia. Notably, HuN1-P230 exhibits a close phylogenetic relationship with several Chinese strains (Figure 4).

### 3.5. Localization of Differential Residues in the E2/E1 Structure

**Figure 5:**
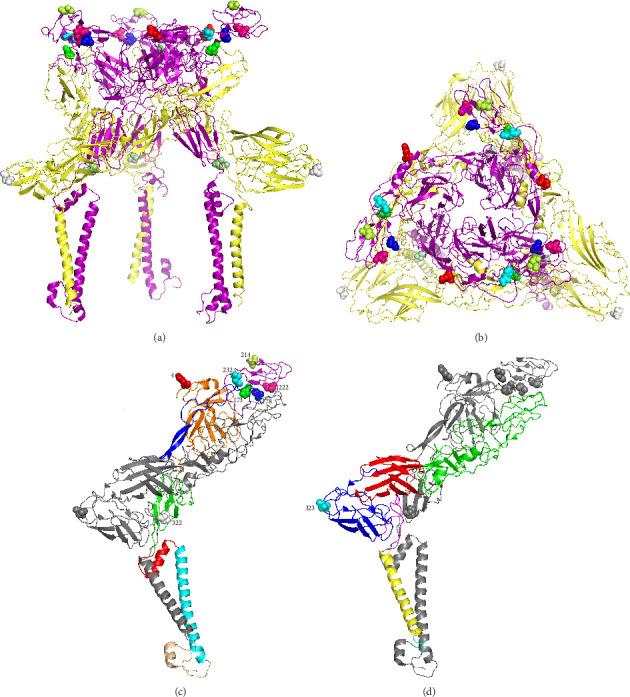
Structural mapping of amino acid variations between HuN1 and HuN1-P230 strains in the GETV spike glycoprotein. (A) Side view and (B) top view of the E1/E2 heterodimer trimer, with E2 shown in purple and E1 in yellow. Mutated residues are depicted as colored spheres. (C) E1/E2 monomer structure showing E2 subdomains: domain A (orange), domain B (purple), domain C (green), domain D (red), β-ribbon (blue), transmembrane helix (cyan), and C-terminal helix (wheat). Mutated residues: 4 (red), 173 (green), 178 (blue), 214 (yellow), 222 (magenta), 232 (cyan), 322 (white). (D) E1 subdomains: domain I (red), domain II (green), domain III (blue), stem-loop (purple), transmembrane helix (yellow), and cytoplasmic tail (cyan), with residue 323 (cyan sphere).

We identified eight amino acid residues that differed between HuN1 and HuN1-P230 within the E1/E2 spike glycoprotein. These residues were mapped onto the Cryo-EM structure of the spike glycoprotein, which forms a trimer composed of E1 and E2 heterodimers (Figure 5A,B). Among these, seven mutations were located in the E2 subunit, while one was found in the E1 subunit (Figure 5A,B).

The E2 subunit consists of an ectodomain (domains A–D and a β-ribbon), a transmembrane helix, and a C-terminal helix (Figure 5C). Residues at positions 173, 178, 214, 222, and 232 are located within domain B and are exposed on the surface of the trimeric spike (Figure 5C). Residue 4 is situated at the apex of domain A and is also surface-exposed, while residue 322 is recessed within a loop between two β-sheets in domain C (Figure 5C). The E1 subunit comprises an ectodomain (domains I–III and a stem-loop), a transmembrane helix, and a cytoplasmic tail (Figure 5D). The mutated residue at position 323 is located in a loop within domain III and is exposed on the molecular surface (Figure 5D).

Notably, our sequence alignment with epidemic GETV strains revealed that all the mutated E1/E2 residues in HuN1-P230 are located in highly conserved regions of the glycoprotein (Figure 3E,F). These structural insights suggest that the observed amino acid substitutions may influence viral virulence by altering surface epitopes or protein interactions.

### 3.6. A Cross-Neutralization Assay of HuN1-P230 and Its Parental Virulent HuN1 Strain

**Figure 6:**
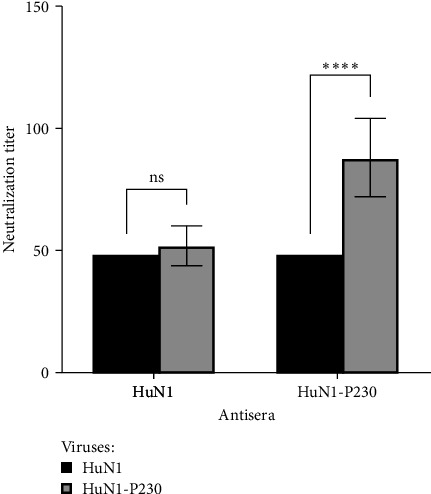
Cross-neutralization of HuN1 and HuN1-P230 strains. Neutralizing antibody titers in antisera against HuN1 and HuN1-P230 were measured using a microtiter assay. Titers are expressed as the reciprocal of the highest serum dilution that inhibited cytopathic effects. Data represent the mean ± standard deviation (SD) from three independent experiments. Statistically significant differences, as assessed by Student's *t*-test, are indicated by asterisks (*⁣*^*∗∗∗∗*^*p* < 0.0001); “ns” denotes not significant.

To evaluate the antigenic relationship between the virulent HuN1 strain and its attenuated variant HuN1-P230, a cross-neutralization assay was performed using mouse immune sera. SN results of the HuN1 immune serum showed that the neutralization antibody titers against HuN1 and HuN1-P230 were broadly similar (Figure 6). However, the HuN1-P230 immune serum exhibited a homologous neutralization titer of 88 ± 16 against the HuN1-P230 strain, compared to a heterologous titer of 48 against the HuN1 strain. This finding indicates that the homologous neutralization titer for the attenuated HuN1-P230 strain is approximately twofold higher than the heterologous titer against the virulent HuN1 strain.

## 4. Discussion

The increasing incidence of GETV outbreaks among mammalian species in recent years underscores its emerging significance as an animal and potential public health threat [20]. This epidemiological trend highlights the urgent need for developing antiviral drugs and effective vaccine candidates [21]. The development of attenuated GETV strains has been achieved through multiple approaches, including reverse genetic engineering, targeted gene deletion, and traditional serial passage attenuation [17, 18, 22]. While reverse genetics enables precise modification of viral genomes and gene deletion offers targeted attenuation, the classical passage attenuation method employed in this study remains a robust approach for vaccine development. Serial passage in cell culture typically selects for viral variants with optimized growth characteristics in vitro, often accompanied by natural mutations that reduce pathogenicity in vivo. In this study, we successfully generated an attenuated GETV strain (HuN1-P230) through 230 serial passages of the virulent HuN1 strain in PK-15 cells. Notably, pregnant mice inoculated with HuN1-P230 at a concentration of up to 10^5^ TCID_50_ showed a 100% survival rate among neonates, while those receiving the parental HuN1 strain developed severe disease manifestations. These findings clearly demonstrate that HuN1-P230 exhibits a significantly attenuated phenotype while maintaining immunogenicity. The observed small-plaque morphology of HuN1-P230, a well-established attenuation marker for alphaviruses [23], further supports its potential as a vaccine candidate.

The molecular determinants of alphavirus pathogenesis involve complex interactions between both structural and nonstructural viral proteins [24–28]. The E2 glycoprotein, as the major component of GETV surface spikes, plays a pivotal role in determining viral virulence [28]. While only the K253R substitution in GETV E2 has been definitively linked to virulence modulation [17], studies of related alphaviruses have identified multiple E2 residues (positions 55, 70, 157, 159, and 243 in Sindbis virus) as critical determinants of neurovirulence [25, 29–31]. Although the HuN1-P230 strain did not contain the exact K253R mutation, seven novel E2 substitutions, with five clustered in domain B (positions 173, 178, 214, 222, and 232) – a region structurally implicated for receptor recognition [12]. The spatial concentration of these mutations in functionally important domains suggests that they may collectively contribute to attenuation through modulation of host cell entry or immune evasion mechanisms.

Beyond structural proteins, emerging evidence highlights the significant role of nonstructural proteins in alphavirus pathogenicity [32]. Particularly, nsP2 mutations (K648A/R649A) have been shown to attenuate GETV by impairing nuclear localization and innate immune evasion [18]. Jiang et al. [22] identified that combinatorial mutations in nsP3's hypervariable domain (HVD) (residues 418–453) and capsid protein (69KPKK72→ 69AAAA72) can attenuate GETV. While HuN1-P230 contains an nsP2 mutation (V650A) previously shown to minimally affect virulence, it harbors potentially significant mutations in other nonstructural proteins. Notably, the nsP1 mutations (positions 214, 220, and 450) are particularly interesting as they map to the membrane-binding, methyltransferase, and guanyltransferase domains, which are crucial for viral RNA capping and replication complex formation [33]. Two nsP3 mutations (G344V and E446V) occur within the recently characterized virulence-determining HVD region [22]. An nsP4 mutation (H175Y) located at the RNA-dependent RNA polymerase domain, which is crucial for viral replication [33]. These findings suggest that attenuation in HuN1-P230 may result from cumulative effects of multiple mutations across both structural and nonstructural proteins, rather than single decisive changes. Future studies employing reverse genetics systems will be essential to dissect the individual contributions of these mutations to the attenuated phenotype.

Remarkably, HuN1-P230 provided complete protection against virulent HuN1 challenge in our immunization studies. The E2 protein's crucial role in eliciting protective immunity [34–36] suggests that the observed surface-exposed mutations may influence immunodominant epitopes. Interestingly, while HuN1-induced antibodies showed comparable neutralization titers against both strains, HuN1-P230 immunization generated significantly higher antibody responses against the homologous strain. This intriguing finding suggests that the attenuation mutations may have altered antige nic properties, potentially enhancing strain-specific immunogenicity. Further studies are needed to characterize these neutralizing epitopes and their protective mechanisms.

## 5. Conclusions

This study demonstrates the successful development of an attenuated GETV strain (HuN1-P230) with an excellent safety profile and protective efficacy. The strain exhibits favorable characteristics for vaccine development, including rapid replication kinetics, high viral titers in cell culture, and complete protection against homologous challenge. Future research should focus on: (1) evaluating genetic stability during serial passage, (2) assessing cross-protection against heterologous GETV strains, and (3) characterizing the precise molecular mechanisms underlying attenuation. These investigations will further validate HuN1-P230's potential as a safe and effective attenuated vaccine candidate for controlling GETV infections in animals and potentially humans.

## Figures and Tables

**Table 1:** Genomic and proteomic variations of different generations of GETV HuN1 strain.

Region	Position		Nucleotide changes				Amino acid changes			
	Nucleotide	Amino acid	HuN1	HuN1- P50	HuN1- P130	HuN1- P230	HuN1	HuN1- P50	HuN1- P130	HuN1- P230
nsP1	718	214	A	T	T	T	T	S	S	S
	736	220	C	C	C	T	R	R	R	C
	1323	—*⁣*^*∗*^	G	A	A	A	—	—	—	—
	1426	450	G	T	T	T	V	L	L	L
	1662	—	A	A	A	G	—	—	—	—

nsP2	1959	—	C	T	T	T	—	—	—	—
	1980	—	T	T	C	C	—	—	—	—
	2017	—	T	C	C	C	—	—	—	—
	3582	—	A	A	G	G	—	—	—	—
	3629	650	T	T	T	C	V	V	V	A

nsP3	5105	344	G	G	G	T	G	G	G	V
	5411	446	A	A	T	T	E	E	V	V
	5430	—	A	A	A	T	—	—	—	—

nsP4	6169	175	C	T	T	T	H	Y	Y	Y

C	7925	—	C	C	C	T	—	—	—	—

E2	8532	4	G	A	A	A	E	K	K	K
	9040	173	C	T	T	T	P	L	L	L
	9055	178	T	T	C	C	I	I	T	T
	9095	—	A	A	C	C	—	—	—	—
	9162	214	G	T	T	T	D	Y	Y	Y
	9187	222	T	T	T	A	I	I	I	K
	9216	232	C	C	T	T	H	H	Y	Y
	9487	322	A	A	T	T	E	E	V	V

E1	10163	—	G	G	A	A	—	—	—	—
	10754	—	C	T	T	T	—	—	—	—
	10938	323	A	A	G	G	N	N	D	D

*Note:⁣* ^*∗*^ Silent mutation.

## Data Availability

The datasets generated and/or analyzed during this study are available from the corresponding author upon reasonable request.

## Supporting Information

Supporting Information The ARRIVE author checklist has been completed and implemented during this systematic review process. Also, the ARRIVE guidelines 2.0 checklist has been used.
